# Clinical evaluation of a topical Unani pharmacopoeial formulation *Tila-e-Kalf* in the management of melasma (*Kalf*): A randomized controlled clinical trial

**DOI:** 10.22038/AJP.2022.21346

**Published:** 2023

**Authors:** Salma, Yasmeen Shamsi, Sadia Nikhat, Mukesh Manjhi, Md. Wasi Akhtar, Sayeed Ahmad

**Affiliations:** 1 *Department of Moalajat, SUMER, Jamia Hamdard, New Delhi, India*; 2 *Department of Ilaj Bil Tadbeer, SUMER, Jamia Hamdard, New Delhi, India*; 3 *Department of Dermatology, HIMSR, Jamia Hamdard, New Delhi, India*; 4 *Department of Pharmacognosy, SPER, Jamia Hamdard, New Delhi, India*

**Keywords:** Chloasma, Hyperpigmentation, Topical treatment, Unani medicine, Hydroquinone

## Abstract

**Objective:**

Melasma is a chronic, acquired, symmetrical hyper melanosis of skin, characterized by irregular light to dark brown patches on sun-exposed areas, with a significant effect on psychological health; melasma is termed as *Kalf* in Unani medicine. Conventional treatments have transitory results and often carry adverse effects like skin irritation, scarring, etc. This study was planned to evaluate the safety and efficacy of a *Unani *pharmacopoeial formulation *Tila-e-Kalf, *comprising of lentil (*Lens culinaris*), bitter almond (*Prunus amygdalus*), and fig (*Ficus carica*), and to compare its efficacy with standard drug hydroquinone in patients of melasma.

**Materials and Methods:**

This was an 8-week open-label, standard controlled, randomized clinical study conducted on patients of epidermal melasma. The test group received *Tila-e-Kalf *while the control group received hydroquinone 4% cream for local application once daily. Efficacy was assessed by MASI (Melasma Area Severity Index), DLQI (Dermatology Life Quality Index), and PGA (Physician Global Assessment) and colored photographs.

**Results:**

Mean MASI score decreased from10.65±0.85 to 7.07±0.74 in the test group (p<0.0001) and from 11.28±1.24 to 7.76±0.9 (p<0.0001) the in control group. Similar improvement was noticed in other parameters also. A large number of patients in the control group reported mild burning, itching, dryness, and skin rashes, while only one patient in the test group reported mild itching.

**Conclusion:**

*Tila-e-Kalf *as a topical depigmenting agent was found equally effective with better tolerability and safety as compared to hydroquinone.

## Introduction

Melasma is a chronic, acquired hypermelanosis of the skin which clinically manifests as irregular brown macules of variable darkness symmetrically distributed on sun-exposed areas of the body particularly on the face (Handel et al., 2014). Pigmented patches may spread onto cheeks, nose, and centre of the forehead, area above eyebrows, upper lips, and chin. (Jagannathan et al., 2017). The word melasma is said to originate from the Greek word ‘*melas*’ which means black, referring to the dark patches on the skin. The disease is also described in ancient literature since the time of Hippocrates (470-360 AD) (Handel et al., 2014). In *Unani* medicine, melasma is known as *Kalf, *and it has been defined as ‘a blackish patch formed by the integration of many black spots called *Barsh’ *(Kabiruddin, 1969).

Melasma is more common in women, accounting for about 90% of all cases, and appears in all races, although it is more common in people with darker skin tones (Ball Arefiev and Hantash, 2012). The prevalence varies between 1.5 and 33.3% in different studies, depending on the population. However, the prevalence rises steeply during pregnancy and has been estimated to be around 50-70% (Shah et al., 2018). Melasma also occurs in men, though it is less common than in women. In an etiological and histological study of Indian males, it was found that men represent 20.5-25.83% of the cases (Sarkar et al., 2014).

It occurs in adults, especially in the age group of 25-45 years. Although melasma does not cause any major health-related complications but severely affects the social life as well as the emotional well-being of the patients. Melasma distresses patients, as it mainly affects the face and is easily visible, and remains constantly present in everyday life, therefore, negatively affecting the quality of life of patients andinfluencing their psychological and emotional well-being (Handel et al., 2014).

Melasma that occurs during pregnancy referred to as “chloasma” may or may not disappear after delivery. It may also occur in non-pregnant ladies and unmarried girls especially if the patient has an associated hormonal imbalance or menstrual irregularities. Genetic factors, excessive exposure to direct sunlight, use of oral contraceptives, pregnancy, and certain drugs or cosmetics may play a role in the pathogenesis of this condition (Niwa et al., 2013). The main drugs implicated in causing skin pigmentation are antimalarial (Aggarwal et al., 2016), amiodarone, cytotoxic drugs, tetracyclines, heavy metals, and psychotropic drugs (Dereure, 2001). Aggravating factors include sun exposure, family history of melasma, pregnancy, and exogenous hormones (oral contraceptives and hormone replacement therapy) (Sadeghpour et al., 2018).

There is no universally accepted and effective therapeutic agent for the cure of melasma. Conventional treatments include topical and oral medications, resurfacing techniques like chemical peeling, light and laser treatments, etc. all of which have produced inconsistent or short-lasting results. Most of these treatments may also lead to skin irritation and further worsening of cutaneous pigmentation or even scarring. Triple-combination cream (TCC) containing hydroquinone, tretinoin, and steroids is currently the only hydroquinone-containing topical therapy approved by the US Food and Drug Administration (US-FDA) for the treatment of melasma (Sadeghpour et al., 2018). However, steroids are known to suppress secretory metabolic products from melanocytes without causing their destruction and therefore have only a short-lived effect on pigmentation disorders (Gupta et al., 2006).

In *Unani* medicine, *Kalf *is considered a melancholic (*sawdawi*) disease that occurs due to the accumulation of black bile (*sawda*) within the skin as a result of leakage from microvasculature (Kabiruddin, 1969; Khan, 2006). It is associated with melancholic (*sawdawi*) disorders of the liver and spleen which lead to the predominance of black bile (*ghalba-e-sawda*) in the blood (Kabiruddin, 1969). Topical treatment in *Unani* medicine includes peeling off of the affected area (Sina, 1992), local application of detergent (*Jali*) drugs followed by astringent (*Qabid*) drugs. Topical application of irritant (*Lazeʻ*) drugs in combination with resolvent (*Muhallil*) drugs is recommended in cases of chronic melasma (Razi, 1986).

*Unani* drugs are reported to be very effective in the management of various dermatological diseases; many herbal drugs have been mentioned to be useful in the treatment of melasma. However, it is necessary to validate these drugs based on scientific parameters through clinical studies. Hence, the present study was planned to evaluate the efficacy of a topical pharmacopoeial formulation *Tila-e-Kalf.*

## Materials and Methods

This was a hospital-based prospective, randomized, standard controlled study conducted on diagnosed cases of melasma (*Kalf*) at Moalajat/ Clinical research OPD, Majeedia Unani Hospital, and Skin OPD, HAHC Hospital, HIMSR, Jamia Hamdard, New Delhi, India

The study protocol was approved by Jamia Hamdard Institutional Ethics Committee, New Delhi. The study was prospectively registered in the Clinical Trial Registry of India, vide Reg. No. CTRI/2019/05/019056, dated 10.05.2019. All the subjects were explained about the trial details, and signed informed consent was taken from them prior to commencement of the trial.

**Inclusion and exclusion criteria**

A total 65 patients of all genders aged 18 years and above with epidermal melasma (which was examined and confirmed by woods lamp) who werewilling to participate in the trial, were included; 10 patients lost to follow-up and a total 55 patients completed the protocol therapy.

The exclusion criteria were:

- Pregnant and lactating women, or those using oral contraceptives and hormonal intrauterine devices.
- Patients treated with tretinoin in the past 3 weeks or treated with hydroquinone in the last 6 months or consumed photosensitizing substances in past 4 weeks
- Patients who hadused any otherbleaching creams or topical steroid creams in the last 4 weeks (Kroon et al., 2011)
- Patients who had undergone any laser therapy and chemical peeling.

A clinical pattern of melasma was assigned to each patient as centrofacial, malar or mandibular by clinical examination. The eligible patients were then randomized by computer-generated randomization chart into the test group (received *Tila-e-Kalf) *and control group (received hydroquinone 4% cream).

**Interventional drugs**

The test formulation is a Unani pharmacopoeial formulation which contains fine powder of lentil (*Lens culinaris*) and bitter almond (*Prunus amygdalus*var.*amara*) in equal proportion. This powder is further mixed with the decoction of fig (*Ficus carica*) in sufficient amount to make a homogenous paste. This prepared paste *“Tila-e-Kalf”* was advised to apply to the affected area of the face once daily at night (Kabiruddin, 2006). The patients of the test group were given 80 g pack of the test drug powder along with a 100 ml decoction of fig. The patients were advised to prepare the paste of the powder with decoction (q.s.) at the time of application.

The patients of the control group were given hydroquinone 4% cream to apply locally over the affected area of the face once daily at night. 

All patients of both the groups were also advised to use sunscreen (UV Blue gel) twice daily (at 8 AM and 1 PM). Patients were advised to apply the cream and pack for 8 weeks.

**Clinical assessment**

At baseline, medical history, facial examination, MASI (Melasma Area Severity Index) Score, colored photograph, DLQI (Dermatology life quality index) score, and PGA (Physician Global Assessment) score were recorded.MASI scores, DLQI score, and PGA were assessed clinically at every follow-up at 2-week intervals (at the 2^nd^ week, 4^th^ week, 6^th^ week, and 8^th^ week). Colored facial photographs were taken at the beginning and on completion of the protocol therapy.

MASI (Melasma Area Severity Index)score is an index used to quantify the severity of melasma and changes during therapy (Kimbrough-Green et al., 1994). The lesions of melasma were assessed and scoring was given; the total scoring was done between 0-48, a higher score shows the greater intensity of the disease. The MASI score is determined by the subjective evaluation of three features viz. A- area of involvement, D- darkness, and H- homogeneity with the (f)- forehead (30%), (rm)- right malar region (30%), (lm)- left malar region (30%), and (c)- chin (10%) of the total face. The area involved in each of these four areas is given a number value of 0 to 6 (0= not involved; 1= <10%; 2= 10-29%; 3= 30-49%; 4= 50-69%; 5= 70-89%; and 6= 90-100%). Darkness and homogeneity are valued on a scale from 0 to 4 (0=absent; 1=slight; 2=mild; 3=marked; and 4=maximum). 

Scoring of MASI: 0.3 A(f) [D(f) + H(f)] + 0.3 A(rm) [D(rm) + H(rm)] + 0.3 A(lm) [D(lm) + H(lm)] + 0.1 A(c) [D(c) + H(c)] (Harumi and Goh, 2016).

PGA (Physician Global Assessment) is the clinical assessment, done at every visit, as 0=Clear, 1= Almost clear, 2= Marked improvement, 3= Moderate improvement, 4= Slight improvement, 5= No improvement, and 6= worse, condition worse than a week 0. 

DLQI (Dermatology Life Quality Index) is calculated by a questionnaire comprising 10 questions. Each answer has a score of 0-3, in total giving a minimum 0 and maximum 30 score; a greater score indicates greater affection of the patient due to the diseased skin condition (Finlay and Khan, 1994).

Documentation of any adverse effects complained by the patients (any redness, itching, inflammation, eruptions, and rashes) was done properly. 

**Safety assessment**

Haemogram, Liver Function Test (LFT), and Kidney Function Test (KFT) were done before treatment and after treatment to assess the safety issue of the test and control regimen.

**Statistical method**

Statistical analyses were performed using InStat software. Wilcoxon matched-pair signed-ranks test (Non-Parametric test) wasadopted for comparison within the group and Mann Whitney U test wasused for comparison of the two groups viz. test and control groups.

## Results

**Patient enrolment**

**Figure 1:**
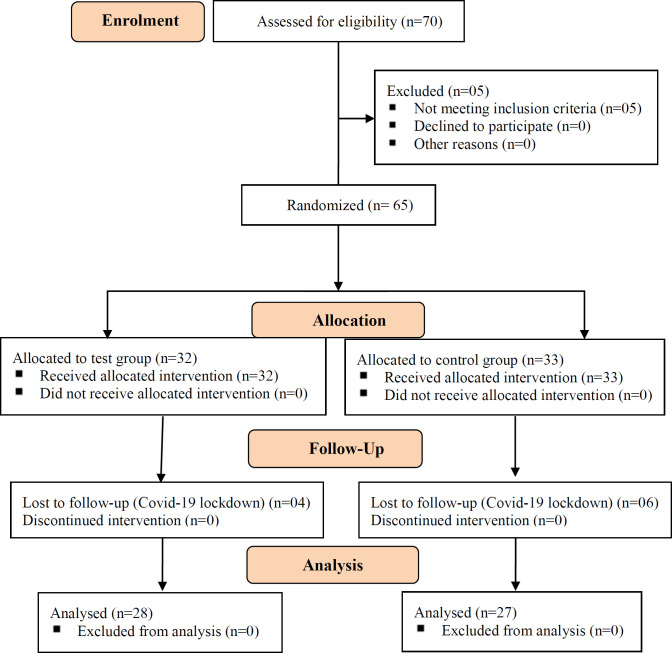
CONSORT flow diagram of the trial

A total of 65 patients (32 in the test group and 33 in the control group) were enrolled in the study after getting the informed consent of each patient. Out of 65 enrolled patients, 55 patients [28 (50.9%) in the test group and27 (49.1%) in the control group] completed the study up to the last visit, i.e. the 8^th^ week of the protocol therapy. Whereas, 10 cases (4 in the test group and 6 in the control group) dropped out due to unknown reasons or could not be followed up because of the Covid-19 pandemic. The details of the patient enrolment and follow-up are mentioned in CONSORT (Figure 1).

**Baseline observations**

In this study, the majority of the patients were found to have malar pattern of melasma, however, in other studies, centrofacial pattern is the most commonly found pattern, followed by the malar pattern. Mandibular is the least found pattern because the cheeks, nose, and forehead are mainly exposed to UV rays (Jain and Balachandrudu, 2018; Zubair et al., 2009). A maximum 29 out of 55 patients were found to have Fitzpatrick type IV skin, indicating that the people having light brown skin are more prone to develop melasma. The baseline and demographic findings are summarized in Table 1.

**Clinical response**

**Figure 2:**
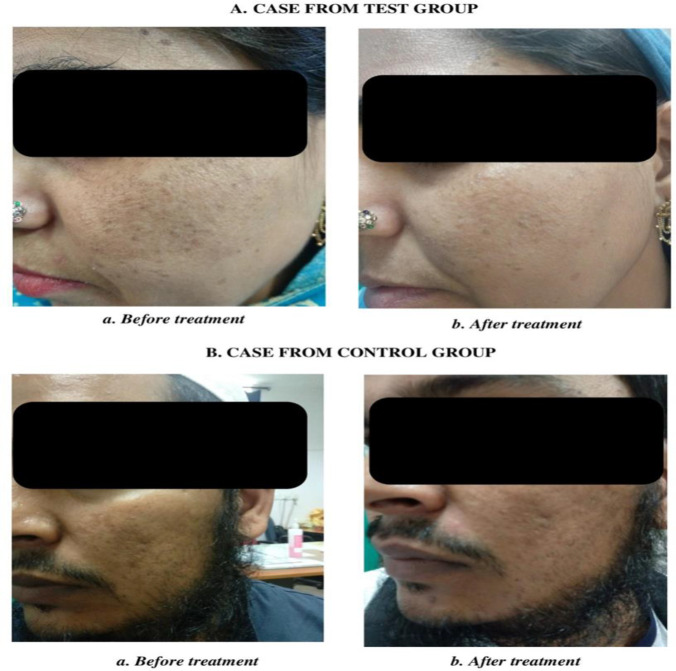
Photographs of melasma patients from Test and Control group

The results showed statistically highly significant improvement in MASI score, PGA score, and DLQI in both test and control groups.On comparing the MASI score, PGA score, and DLQI score of test group with control group, statisticallyno significant change was found (Table 2). Colored photographs of all patients before and after treatment also showed significant improvement in the hyperpigmentation of facial skin (Figure 2).

**Table 1:** Baseline observations

**Patient characteristics**	**No. of patients in test group (n=28)**	**No. of patients in control group (n=27)**	**Total no. of patients (n=55)**
**Age group**			
20-25 years 26-30 years	2 7	3 6	5 13
31-35 years 36-40 years	4 4	3 10	7 14
41-45 years 46-50 years	5 5	3 2	8 7
51-55 years	1	0	1
**Gender** Male Female	2 26	4 23	6 49
**Marital status** Married Unmarried	25 3	23 4	48 7
**Fitzpatrick skin type** I II III IV V VI	0 1 10 14 3 0	0 0 8 15 3 1	0 1 18 29 6 1
**Site of lesion** Centrofacial Malar Mandibular	3 25 0	3 24 0	6 49 0
**Duration of complaints** 0-5 years 6-10 years 11-15 years	20 6 2	19 6 2	39 12 4

**Side/ adverse effects**

The test and control drugs were found almost equally effective in reducing the melasma. However, a significant number of patients in the control group reported side/ adverse effects, like redness, dryness, itching/ burning sensation. The majority of adverse events were mild, and hence, none of the patients discontinued treatment due to side effects. Also, 30% patients of the control group reported dryness and 44% patients reported itching at the 2^nd ^week. Erythema was reported by almost 15% patients of in the control group in the 4^th^ week, which was slightly decreased in the 8^th^ week. The reported adverse effects in the patients of the control group indicated that hydroquinone has these adverse effects (Khosravan et al., 2017); whereas, in the test group, only one patient reported mild itching, which recovered after a few days. 

The safety parameters viz. CBC (Complete Blood Count), LFT (Liver Function Test), and KFT (Kidney Function Test) were within their normal limits in both groups. In all these parameters, the difference was statistically insignificant before and after the treatment in both groups (Table 3). Therefore, these findings showed that the treatments were completely safe in both groups.

## Discussion

The treatment of melasma has always been challenging due to its recurrent and refractory nature. As for the treatment, till date, there is no universally acceptable effective drug/ formulation which can cure this disease permanently. This study showed that both hydroquinone 4% and *Tila-e-Kalf* (an Unani pharmacopoeial polyherbal formulation) are almost equally effective on currently used scientific efficacy parameters. Hydroquinone 4% is considered a standard drug for melasma. The study conducted by Khosravan et al. (Khosravan et al., 2015) demonstrated a positive response with hydroquinone similar to our study.

The effect produced by the test drug formulation may be attributed to the different medicinal effects of its constituent ingredients.

**Table 2:** Results of outcome measures before and after treatment in both groups (mean±SEM)

**Parameter**	**Test group (n=28)**		**Control group (n=27)**	
	**BT**	**AT**	**BT**	**AT**
MASI	10.65±0.85	7.07±0.74	11.28±1.24	7.76±0.9
	Wilcoxon test (Non-Parametric test) p<0.0001		Wilcoxon test (Non-Parametric test) p<0.0001	
	Mann-Whitney U-test BT: p=0.4426; AT: p=0.4425			

PGA	4.75±0.083	3.60±0.118	4.962±0.037	3.814±0.076
	Wilcoxon test (Non-Parametric test) p<0.0001		Wilcoxon test (Non-Parametric test) p<0.0001	
	Mann-Whitney U-test BT: p=0.137; AT: p=0.486			

DLQI	17.10±1.03	13.25±0.77	18.66±0.68	15.33±0.57
	Wilcoxon test (Non-Parametric test) p<0.0001		Wilcoxon test (Non-Parametric test) p<0.0001	
	Mann-Whitney U-test BT: p=0.1513; AT: p=0.1738			

Within the group Wilcoxon test (Non-Parametric test) was applied; highly significant change was observed within the group (p<0.0001).

Between the groups comparison was done by applying Mann Whitney U test; no significant change was observed (p>0.05).

**Table 3:** Safety measures before and after treatment in both groups (mean±SD)

Safety parameters	Test group (n=28)			Control group (n=27)		
	BT	AT	p*-*value (Paired t-test)	BT	AT	p*-*value (Paired t-test)
Haemogram						
Hb%	11.82±1.5	11.73±1.0	0.371	12.12±1.4	12.18±1.5	0.410
TLC (per/cmm)	7494.2±1327.7	7696.4±1720.6	0.118	7162.9±268.7	7259.2±332.5	0.4005
Neutrophils %	59.17±7.75	60.10±7.80	0.0614	61.47±6.85	61.88±5.05	0.3691
Lymphocytes %	32.6±7.457	32.4±7.089	0.0624	32.2±6.765	32.4±6.469	0.2955
Eosinophils %	3.11±1.39	3.31±1.26	0.1570	3.25±1.47	3.15±0.89	0.3442
Monocyte %	2.39±1.25	2.14±0.89	0.1147	2.15±1.13	1.85±0.53	0.1328
Platelets (L/cmm)	2.12±0.14	1.95±0.09	0.0685	2.32±0.71	2.17±0.66	0.1200
RBC count (M/ cmm)	4.22±0.58	4.13±0.36	0.1671	4.07±0.52	4.08±0.52	0.4759
ESR (mm/ 1Hr)	12.64±6.8	13.60±7.0	0.0659	6.02±3.6	6.66±2.3	0.1080
LFT						
S. Bilirubin (mg/dl)	0.68±0.11	0.68±0.11	0.480	0.701±0.10	0.718±0.08	0.253
SGOT (IU/L)	34.96±9.42	34.03±6.58	0.332	35.11±11.06	37.44±8.64	0.128
SGPT (IU/L)	37.35±11.31	37.21±6.97	0.476	36.14±11.61	36.96±9.05	0.136
Alk. phosphatase (IU/L)	143.07±26.07	135.89±17.98	0.106	134.00±28.71	129.92±22.26	0.060
KFT						
Blood urea (mg/dl)	22.32±5.28	21.71±4.47	0.3003	22.70±5.11	21.14±2.74	0.0941
S. Creatinine (mg/dl)	0.82±0.17	0.82±0.12	0.2458	0.82±0.19	0.81±0.13	0.2575
Uric acid (mg/dl)	4.69±1.03	4.21±0.85	0.0227	4.85±0.72	3.98±0.73	0.4050
Total protein (g/dl)	7.09±0.36	7.00±0.16	0.1140	6.94±0.33	6.95±0.22	0.4064
Albumin (g/dl)	3.69±0.32	3.77±0.25	0.4688	3.78±0.33	3.85±0.73	0.3373
Globulin (g/dl)	3.13±0.27	3.19±0.19	0.1508	3.18±0.22	3.14±0.16	0.1398

Fig has detergent (Jali) effect and is used in various skin diseases like melasma (Kalf) (Kabiruddin, 1951; Khan, 2012). The fruit of fig contains numerous bioactive compounds; 2 groups of phenolic compounds are mainly present which are phenolic acids (syringic acid, gallic acid, and chlorogenic acid) and flavonoids (anthocyanins, catechin, and epicatechin). Flavonoids in the fig fruit are accountable for their great antioxidant property (Ali et al., 2012). In melasma, melanin synthesis is increased and tan produced by dendritic melanocytes is disseminated at the dermo-epidermal junction. The main enzyme responsible for the production of melanin is tyrosinase. Fig contains tyrosinase inhibitors which work as a skin depigmenting agent. The decline in melanin production in the skin can be mainly credited to gallic acid and catechins present in it. The high content of vitamin C (ascorbic acid) in fig is well-known to stimulate and increase collagen biosynthesis in the dermis, thus improving its hydration level. The extract of fig fruit has a good effect on transepidermal loss, hydration values, and sebum content, and thus have a therapeutic effect on melisma (Akhtar et al., 2014).

Lentil (*Lens culinaris*) is a leguminous plant that contains soyasaponins which are the bioactive secondary plant metabolites. Soyasapogenol A impedes melanogenesis via down-regulating the protein levels of TRP-1 and TRP-2, which results in reduced melanin production in the skin. Therefore, soyasapogenol A is effective in treating the hyperpigmentation disorders like melasma, frecklesetc., and is considered an effective skin whitening agent that may also be used in fairness cosmetics (Yang et al., 2019). According to *Unani* medicine, the lentil has a detergent (*Jali*) action in skin ailments and improves the complexion of the skin. The topical use of lentils regularly for a longer duration reduces black spots on the face (Baitar, 1999; Khan, 2014).

Bitter almond contains various nutrients, vitamins, minerals, flavonoids, and phenolic compounds. These phenolic compounds and flavonoids have a strong antioxidant property. Vitamin E is present in abundance in bitter almonds, which also acts as a strong antioxidant and is used for rejuvenation of skin and various skin disorders (Moradi et al., 2017). In *Unani* system of medicine, bitter almond is recommended for various skin disorders like melasma (*Kalf*) and freckles (*Barsh*)*. *Bitter almond topically acts as a detergent (*Jali*), which improves skin complexion and works on hyperpigmentation as well. Therefore, bitter almond is a drug of choice for the treatment of melasma for local application in such cases (Khan, 2012).

In the present study, both hydroquinone 4% and *Tila-e-Kalf*(Lentil, Bitter almond, and Fig) appeared equally effective on different efficacy parameters.

The majority of adverse events were reported by patients in the control group, whereas, the test drug was found more tolerable.

The safety and efficacy profile obtained in this study indicate that the test drug *Tila-e-Kalf*can be used for an extended duration effectively as compared to the most prescribed conventional drug hydroquinone in the treatment of melasma (*Kalf*).

## Conflicts of interest

The authors have declared that there is no conflict of interest.
